# Supplementary material for: Mechanistic Pathways and Molecular Targets of Plant-Derived Anticancer ent-Kaurane Diterpenes
Source: Biomolecules. 2020 Jan 16;10(1):144. doi: 10.3390/biom10010144 (PMC7023344; doi:10.3390/biom10010144)
Supplement: Supplementary file 1 [file biomolecules-10-00144-s001.pdf]

Table S1. Plant-derived anticancer *ent*-kaurane diterpenoids with their biological targets.

Compounds	Plant source	Cytotoxicity				Targets	References
		IC_{50} (μM)	Cell line	Cancer cell types	Incubation time		
Oridonin	*Isodon rubescens*	32.6	HCT-116	Colorectal	24 h	GLUT1, MCT1, LC-I, LC-II, p-AMPK, caspase-3, -8, -9, cytochrome c, BCL-2, BAX, p53, p21, PARP, BCL-X_L, EGF, CDK1, PI3K, p-Akt, VEGFA, VEGFR2, VEGFR3, claudin-1, -4, and -7, NF-κB, IκB-α, notch1-4	[16-32]
		3.27	OCI-AML3	Myeloid leukemia			
		53.0	BxPC-3	Pancreatic			
		28.7	KYSE-150	Esophageal	48 h		
		34.4	EC9706	Esophageal			
		32.3	KYSE-30	Esophageal			
		0.73	Jurkat cells	Leukemia			
		10.9	MG-63	Osteosarcoma			
		11.9	HOS	Osteosarcoma			
		17.3	Saos-2	Osteosarcoma			
		17.7	U-2OS	Osteosarcoma			
		19.7	EC109	Esophageal			
		31.3	EC9706	Esophageal			
		25.8	EC1	Esophageal			
		420	HUVEC	Endothelial			
		3.66	CNE1	Nasopharyngeal			
		5.93	CNE2	Nasopharyngeal			
		67.1	EC109	Esophageal	72 h		
		53.5	SHG-44	Esophageal			
		72.1	MCF-7	Breast			

		22.7	SGC-7901	Gastric	72 h		
		1.65	CCRF-CEM	Leukemia			
		8.53	CEM/ADR5000	Leukemia			
		6.06	MDA-MB231	Breast			
		9.74	MDA-MB231/ BCRP	Breast	-		
		18.0	HCT116 (p53+/+)	Colorectal			
		34.7	HCT116 (p53-/-)	Colorectal			
		17.4	U87MG	Glioblastoma			
		25.7	HepG2	Liver			
		>109	AML12	Normal			
Eriocalyxin B	*I. eriocalyx* var. laxiflora	0.76	SMMC-7721	Liver		Cyclin D1, CDK4, p-Rb, p-VEGFR-2, pTyr^{1175}-VEGFR-2 pTyr^{1213}-VEGFR-1, pTyr$^{576-577}$-FAK, pTyr^{416}-Src, pSer^{473}-Akt, pThr^{202}/Tyr^{204}-ERK1/2, pThr^{180}/Tyr^{182}-p38-MAPK, LC3B-II, beclin-1, p62, cleaved caspase-3, cleaved PARP, BCL-2, p-Akt, p-mTOR, p-p70S6K, p-JAK2, p-STAT3, VEGF, VEGFR-2, MMP-2,	[33-39]
		0.75	MCF-7	Breast	48 h		
		0.47	MDA-MB-231	Breast			
		1.79	PANC1	Pancreatic			
		0.86	CAPAN1	Pancreatic			
		0.73	CAPAN2	Pancreatic			
		1.40	SW1990	Pancreatic	72 h		
		>3.58	WRL68	Normal			
		>5.83	PBMC	Normal			
		1.00	SU-DHL-4	Lymphoma			
		1.50	Namalwa	Lymphoma			
		2.00	Raji	Lymphoma	72 h		

		2.00	Jurkat	Lymphoma		MMP-9, PCNA, NF-κB p50	
		5.60	U266 cells	Lymphoma			
		2.50	HUT78 cells	Lymphoma			
Excisanin A	*I. macrocalyxin*	22.4	MDA-MB-231	Breast	72 h	MMP-2, MMP-9, integrin β1, β-catenin, p-FAK, p-Src, p-JNK, p-c-Jun, sestrin 2, p-PI3K, p-Akt, p-GSK3β, p-GSK-3α, Thr308-Akt, Ser473-Akt, p-mTOR and p-FKHR	[40-42]
		27.3	SKBR3	Breast			
		10.3	MDA-MB-453	Breast			
		6.45	Hep3B	Hepatoma			
Ponicidin	*I. rubescens, I. japonicas*	23.1	HeLa cells	Cervical	24 h	Bcl-2, p-JAK2, p-STAT3, BAX, cleaved caspase-3, -8, -9, p-ERK, p-Akt, p-p38	[43-46]
		38.0	A549 cells	Lung			
		32.0	GLC-82	Lung			
		31.0	A549 cells	Lung	48 h		
		26.0	GLC-82	Lung			
		15.0	A549 cells	Lung	72 h		
		13.0	GLC-82 cells	Lung			
Pharicins A and B	*I. pharicus*	~3.50	NB-4, U937, THP-1	Leukemia	48 h	BubR1, RAR-α	[47, 48]
Jaridonin	*I. rubescens*	12.0	EC109	Esophageal	48 h	Cleaved caspase-3, -9, cytochrome c, p53, BAX, p21waf1/Cip1, p-ATM (Ser1981), p-Cdc2 (Tyr15), p-Cdc25C, CHK1, CHK2 and	[23, 26, 49]
		11.2	EC9706	Esophageal			
		4.60	EC1	Esophageal			
		14.7	SHG-44	Glioma	72 h		
		16.7	MCF-7	Breast			

						p-H2A.X (Ser139)	
Jungermannenone A	*Jungermannia fauriana*	1.34	PC3	Prostate	24 h	c-myc, cyclin D1, cyclin E, CDK4, p-Cdc2, p21, pERK, Ku70/Ku80, RDA51	[50]
		5.01	DU145	Prostate			
		2.78	LNCaP	Prostate			
		8.64	A549	Lung			
		18.3	MCF-7	Breast			
		5.29	HepG2	Liver			
		5.09	RWPE1	Normal			
Jungermannenone B		4.93	PC3	Prostate			
		5.50	DU145	Prostate			
		3.18	LNCaP	Prostate			
		5.26	A549	Lung			
		14.2	MCF-7	Breast			
		6.02	HepG2	Liver			
		18.2	RWPE1	Normal			
Effusanin E	*I. serra*	~60	CNE2	Nasopharyngeal	48 h	Cleaved PARP, caspase-3, -9, p65 NF-κB, p50 NF-κB, COX-2	[51]
		Non-toxic up to 500 μM	RWPE1	Normal			
Longikaurin A	*I. ternifolius*	~1.8	SMMC-7721	Liver	48 h	Skp2, p21, p-Cdc2 (Try15), cyclin B1, Cdc2, p-JNK, c-myc, fibronectin, cleaved caspase-3, PARP, BAX, BCL-XL, p-Akt and p-GSK-3β	[25,52,53]
		~2	HepG2	Liver			
		~6	BEL7402	Liver			
		~6	Huh7	Liver			
		~9	LO2	Liver			
		1.26	CNE1	Nasopharyngeal			

		1.52	CNE2	Nasopharyngeal			
Glaucocalyxin A	*I. japonica*	6.15	HL-60	Leukemia	24 h	Caspase-3, -9, p-AKT, p-Bad, BAX, BCL-2, XIAP, cytochrome c, PARP, p53, Fas, FasL, p-ERK, p-JNK, PTEN, LC3 II/I	[54-62]
		2.70	Focus	Liver	48 h		
		5.58	SMMC-7721	Liver			
		8.22	HepG2	Liver			
		2.87	SK-HEP1	Liver			
		7.02	HOS	Osteosarcoma			
		7.32	Saos-2	Osteosarcoma			
		8.36	U-2OS	Osteosarcoma			
		5.30	MG-63	Osteosarcoma			
		9.77	UMUC3	Bladder			
		1.00	MCF-7	Breast	72 h		
		4.00	Hs578T	Breast	72 h		
Glaucocalyxin B		5.86	HL-60	Leukemia	24 h		
		13.4	SGC-7901	Gastric	60 h		
		4.61	HeLa	Cervical	72 h		
		3.11	SiHa	Cervical			
Lasiodin	*I. serra*	~6	CNE1	Nasopharyngeal	24 h	Apaf-1, cytochrome-C, cleaved PARP, caspase-3, caspase-9, p-Akt, p-ERK1/2, p-p38, p-JNK, COX-2, NF-κB	[63]
		~5	CNE2	Nasopharyngeal			
Adenanthin	*I. adenanthus*	2.31	HepG2	Liver		Peroxiredoxin I and II, p-ERK, p-c-Jun, C/EBPβ	[26, 64, 65]
		6.67	Bel-7402	Liver			
		8.13	SMMC-7721 cells	Liver			

		19.6	Human immortal hepatic cell lines QSG-7701	Normal	48 h		
		20.4	HL-7702 cells	Normal			
		6.50	EC109	Esophageal	72 h		
		4.80	SHG-44	Glioma			
		7.60	MCF-7	Breast			
Kaurenic acid	*Espeletia semiglobulata*	0.79	B16F1	Melanoma	-	BCL-X_L, p53, c-myc, CCND1, BCL-2, caspase-3, ATM, CHK2 and TP53	[66-68]
Weisiensin B	*I. weisiensis*	10.0	BEL-7402	Liver	48 h	-	[69, 70]
		3.24	HepG2	Liver			
		32.0	HO-8910	Ovarian			
		4.34	SGC-7901	Gastric			
Inflexinol	*I. excisus*	29.0	SW620	Colorectal	48 h	BCL-2, XIAP, cIAP1/2, cleaved caspase-3, -9, PARP, cyclin D1, NF-κB p50, p65, IκB,	[71]
		30.0	HCT116 (p53+/+)	Colorectal			
		34.0	HCT116 (p53−/−)	Colon			
		Non-toxic up to 40 μM	Normal CCD-112 CoN cells	Normal			
Xerophilusin B	*I. xerophilus*	2.80	KYSE-140	Esophageal	72 h	Cytochrome c, cleaved caspase-3, -7, -9, PARP, BCL-2, BAX	[72]
		1.20	KYSE-150	Esophageal			
		1.70	KYSE-450	Esophageal			

		2.60	KYSE-510	Esophageal			
Henryin	*I. rubescens*	0.27	SW480	Colorectal	72 h	Cyclin D1, c-myc, β-catenin, TCF4	[73]
		0.77	HT-29	Colorectal			
		0.90	HCT-116	Colorectal			
		2.47	A549	Lung			
		2.98	Normal colon cells CCD-841-CoN	Normal			
		3.55	Normal bronchus cells BEAS-2B	Normal	72 h		
EPLE	*Salvia cavaleriei*	-	HCT-116 and SW480	Colorectal	-	c-myc, axin2, survivin, β-catenin, BCL-2, Bcl-xL, Bim, caspase-3	[74]
DEK	*Rubus corchorifolius*	40.0	HCT-116	Colorectal	72 h	Cleaved caspase-3, -9, PARP, p53, BAX, p21Cip1/Waf1, cyclin D1, CDK2, CDK4, EGFR, COX-2, Akt	[75]
		Non-toxic up to 100 μM	Human colonic myofibroblasts CCD-18-Co	Normal			
JDA-202	*I. rubescens*	8.60	EC109	Esophageal	24 h	Peroxiredoxin I, p-JNK, p-p38, p-ERK	[26]
		9.40	EC9706	Esophageal			
		36.1	HET-1A	Normal			
		26.2	KYSE-450	Normal			
Pterisolic acid G	*Pteris semipinnata*	20.4	HCT-116	Colorectal	24 h	Dvl-2, GSK-3β, β-catenin, cyclin D1, c-myc, p53, puma,	[76]
		16.2	HCT-116		48 h		

		4.07	HCT-116		72 h	cleaved PARP, cleaved caspase-3, p-p65, BCL-2, BCL-X_L	
Rabdoternin B	-	23.2	SW480	Colorectal	48 h	-	[77-79]
		36.3	HT-29	Colorectal			
		20.7	HCT-116	Colorectal			
		>40	CCD-841-CoN	Normal			
Maoecrystal I	*I. xerophilus*	16.2	SW480	Colorectal	48 h	c-myc, cyclin D1, survivin and axin2	[77-79]
		11.4	HT-29	Colorectal			
		26.2	HCT-116	Colorectal			
		>40	CCD-841-CoN	Normal	48 h		
CHKA	*Wedelia chinensis*	-	HUVECs	Endothelial cells	-	p-VEGFR-2, p-mTOR, p-Akt and p-ERK	[80]
CrT1	*Croton tonkinensis*	8.40 to 31.2	Various cancer cells	-	-	Caspase-3, -7, -8, -9, PARP, p53, BAX, BCL-2, cytochrome c, p-AMPK, p-mTOR, p70S6K	[81]
DHK	*C. malambo*	40.8	MCF-7	Breast	72 h	BCL-2, hTERT, Ap2α–Rb, E2F1	[82, 83]
KD	*Jungermannia truncata*	0.56	HL-60	Leukemia	12 h	Caspase-8, -9, Bid	[84]
OZ	*Parinari* spp.	5.00	Molt4	Leukemia	48 h	PARP, caspase-3, hypodiploidia, phosphatidylserine	[85]
